# A significant net sink for CO_2_ in Tokyo Bay

**DOI:** 10.1038/srep44355

**Published:** 2017-03-13

**Authors:** Atsushi Kubo, Yosaku Maeda, Jota Kanda

**Affiliations:** 1 Department of Ocean Sciences, Tokyo University of Marine Science and Technology, 4-5-7 Konan, Minato-ku, Tokyo, 108-8477, Japan

## Abstract

Most estuaries and inland waters are significant source for atmospheric CO_2_ because of input of terrestrial inorganic carbon and mineralization of terrestrially supplied organic carbon. In contrast to most coastal waters, some estuaries with small freshwater discharge are weak source or sometimes sink for CO_2_. Extensive surveys of pCO_2_ in Tokyo Bay showed that the overall bay acts as a strong net sink for atmospheric CO_2_. Although small area was a consistent source for CO_2_, active photosynthesis driven by nutrient loading from the land overwhelmed the CO_2_ budget in the bay. Here we show a comprehensive scheme with a border where air-sea CO_2_ flux was ±0 between nearshore waters emitting CO_2_ and offshore waters absorbing CO_2_. The border in Tokyo Bay was extremely shifted toward the land-side. The shift is characteristic of highly urbanized coastal waters with an extensive sewage treatment system in the catchment area. Because highly urbanized coastal areas worldwide are expected to quadruple by 2050, coastal waters such as Tokyo Bay are expected to increase as well. Through extrapolation of Tokyo Bay data, CO_2_ emission from global estuaries would be expected to decrease roughly from the current 0.074 PgC year^−1^ to 0.014 PgC year^−1^ in 2050.

Despite their relatively small areal coverage, coastal waters significantly contribute to the global account of CO_2_ exchange due to their dense biological activities^1^,^2^,^3^. Coastal waters have been regarded as CO_2_ sources to the atmosphere because of their input of terrestrial organic carbon and subsequent mineralization^2^,^3^. However, current estimates of the coastal CO_2_ budget are based on observations in only limited types of marine systems, irrespective of the large heterogeneity in such biogeochemical settings. In fact, Kuwae *et al*.^4^ have recently discussed the possibility of net carbon fixation in certain types of coastal systems including those affected by intense anthropogenic activities. Furthermore, the available observational data from a particular system are generally insufficient to cover the large spatial and temporal variability of the coastal carbon cycle, thus resulting in an uncertain estimate of the carbon budget. More data from various systems with sufficient areal and temporal coverage are thus needed.

We conducted extensive observations of the partial pressure of carbon dioxide (pCO_2_) in Tokyo Bay from March 2007 to December 2010 during 49 observational cruises. Tokyo Bay is a semi-enclosed embayment with an area of approximately 1320 km^2^ and a mean water depth of 19 m. The bay is bounded by highly urbanized areas. The bay has suffered from severe cultural eutrophication since the late 1950 s, along with rapid development in catchment areas including the Tokyo metropolis. Sewer systems with secondary or tertiary treatment cover most of the urban regions, and yet phytoplankton blooms persist throughout the year and anoxic bottom waters consistently appear during the summer. In some estuary, carbonate dissolution contribute to coastal CO_2_ concentration about 20%^5^. As regard to Tokyo Bay, shellfish at the bottom water are the major calcifier and calcifying plankton rarely dominates^6^. In addition, surface waters at the bay are oversaturated with respect to calcium carbonate saturation throughout the year^7^. Therefore, we suppose that carbonate dissolution do not contribute to coastal CO_2_ in the bay.

## Results and Discussion

**Figure 1:**
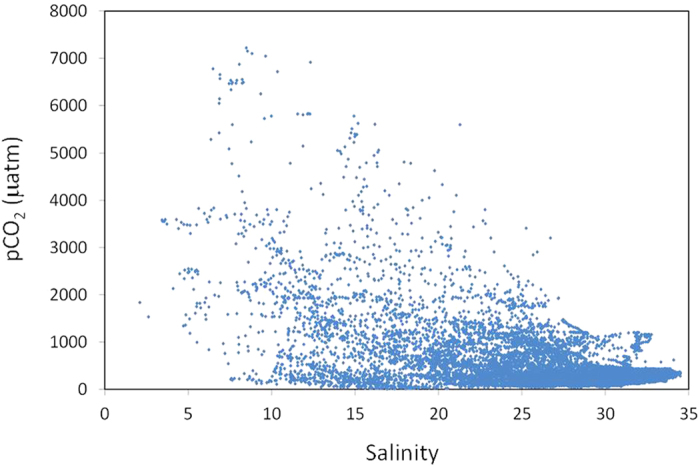
All pCO_2_ (μatm) data points (n = 21,706) plotted against salinity.

All of the pCO_2_ data points (n = 21,076) from our observations are plotted against salinity in Fig. 1, and each point in the figure represents the value of pCO_2_ at an interval of one minute during the observation. R/V Seiyo-maru observed most of the area during 40 cruises from March 2007 to December 2010 on a monthly basis. R/V Hiyodori observed the innermost head of the bay during nine cruises during the period from May 2009 to October 2010. The pCO_2_ values of surface bay ranged from 10 to 7218 μatm. The pCO_2_ values for 21,076 data indicated that a total of 16,345 points were under-saturated with respect to the atmospheric equilibrium; oversaturation was found only in 4,731 data points.

**Figure 2:**
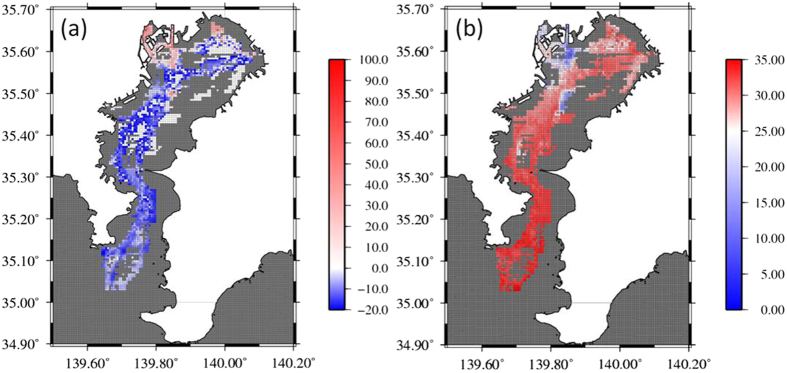
Map of (**a**) sea-air flux (mmol C m^−2^ day^−1^) and (**b**) salinity, binned into a 500 m × 500 m horizontal resolution grid. Positive flux values (red) represent outgassing of CO_2_ to the atmosphere, and negative values (blue) represent uptake. Maps were created using Generic Mapping Tools software (GMT v4.5.12; http://gmt.soest.hawaii.edu/)^37^.

The air-sea CO_2_ fluxes were calculated from all of the pCO_2_ data (see Methods). The results obtained from different times were binned into a 500 m × 500 m horizontal resolution grid and then averaged together (Fig. 2a). The northwestern head of the bay was the only area with consistent positive CO_2_ flux to the atmosphere, whereas the central bay and bay mouth were a sink for atmospheric CO_2_. The location where CO_2_ flux was ± 0 roughly coincided with the location where the average annual surface salinity was 25 (Fig. 2b). The areas where salinity was above and below 25 were estimated according to methodology described in Ninomiya *et al*.^8^. The average CO_2_ flux (positive values indicate efflux into the air) for areas where salinity was less than 25 (81 km^2^) was 15.2 mmolC m^−2 ^day^−1^. In contrast, the average CO_2_ flux for areas where salinity was greater than 25 (1239 km^2^) was –10.6 mmolC m^−2 ^day^−1^. The area weighted annual CO_2_ flux in Tokyo Bay had a rate of –8.8 mmolC m^−2 ^day^−1^; the bay as a whole was a strong net sink for atmospheric CO_2_. The annual CO_2_ flux in Tokyo Bay was calculated to be –5.2 × 10^10^ gC year^−1^.

**Figure 3:**
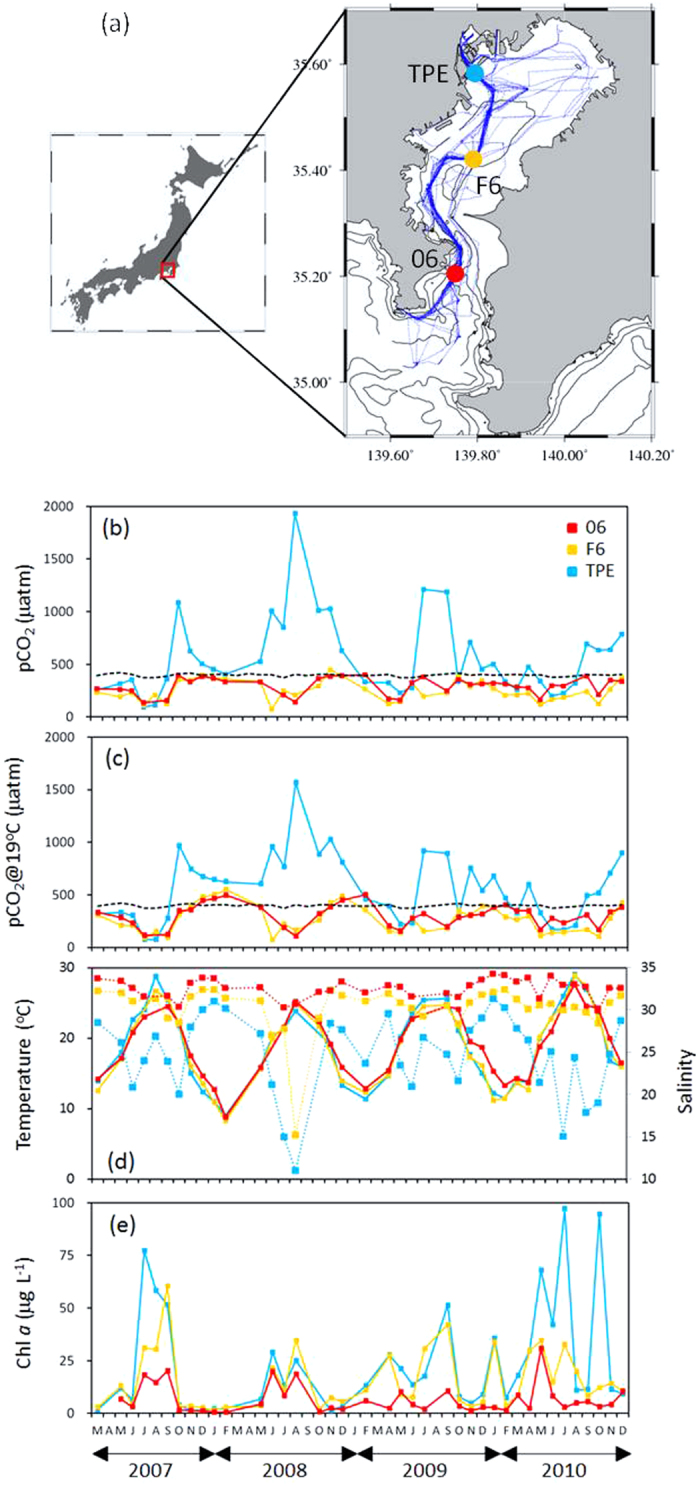
(**a**) Map of the study site. The ship routes of all cruises are shown as blue lines. Data were measured with a sampling frequency of one minute (n = 21076). Circles indicate the three representative stations (06, F6, and TPE). TPE stands for Tokyo Port Entrance. Isobaths of 20-, 50- and 200-m are also shown. (**b**) Seasonal variations of pCO_2_ (μatm) and atmospheric pCO_2_ (the dotted line). (**c**) pCO_2_ normalized to average temperature (19 °C). (**d**) Temperature (°C) and salinity and (**e**) Chl *a* (μg L^−1^) at the three representative stations during the observation period (March 2007 – December 2010). Map (**a**) was created using Generic Mapping Tools software (GMT v4.5.12; http://gmt.soest.hawaii.edu/)^37^.

The seasonal variations of pCO_2_ and related parameters at three representative stations in the bay are presented in Fig. 3. The parameters include the observed pCO_2_, pCO_2_ normalized to average temperature (19 °C), temperature, salinity and chlorophyll *a* (Chl *a*) concentration. In the northwestern head of the bay (St. TPE), where salinity was lowest among the three stations, pCO_2_ values generally exceeded the atmospheric equilibrium. There was no distinct pattern observed for the seasonal variation of pCO_2_ (93–1920 μatm), and the variation of pCO_2_ was not correlated with Chl *a* (R^2^ = 0.03, *P > *0.1, n = 39). At the central bay (St.F6) and the bay mouth (St.06), low values of pCO_2_ were observed during spring and summer (70–336 μatm), whereas pCO_2_ values were close to atmospheric equilibrium values during autumn and winter (264–449 μatm). In terms of the seasonal variation of pCO_2_ in the central bay and bay mouth, a negative correlation was found between pCO_2_ and Chl *a* (R^2^ = 0.64, *P < *0.001, n = 77). The correlation suggests that active photosynthesis reduced pCO_2_ during spring and summer, hence suggesting that pCO_2_ in surface waters is mainly controlled by biological activity. The stratified water column prevents the CO_2_ supply from the deeper waters with high CO_2_ levels. In autumn and winter, the low photosynthetic rate along with the well-mixed water column resulted in pCO_2_ values close to the atmospheric equilibrium. Temperature did not significantly contribute to the seasonal variation of pCO_2_. The pCO_2_ values normalized to annual average temperature (19 °C), which accounted for the temperature dependence of equilibrium constants and the solubility coefficient^9^, did not affect the seasonal variation of pCO_2_.

In June 2010, pCO_2_ decreased to a low of 10 μatm, and Chl *a* concentration was >300 μg L^−1^, which we found to be the lowest reported value in a marine environment. Based on the low observed CO_2_ concentrations, the exhaustion of gaseous CO_2_ in seawater resulted in the limitation of the CO_2_ supply to algal cells, which may limit the cells growth. In such CO_2_ limiting conditions, phytoplankton would need to take up bicarbonate using the proton pump mechanism and the carbonic anhydrase^10^.

The CO_2_ absorption of 5.2 × 10^10^ gC year^−1^ in Tokyo Bay was in accordance with the overall carbon budget of the bay. A mass balance model estimated the total organic carbon (TOC) influx from the rivers to Tokyo Bay to be 8.1 × 10^10^ gC year^−1^ and the TOC efflux from the bay to the open ocean to be 9.4 × 10^10^ gC year^−1^ 11. On the basis of actual observations, the amount of organic carbon burial was estimated to be 4.2 × 10^10^ gC year^−1^ 12. A different box model estimated the dissolved inorganic carbon (DIC) influx to Tokyo Bay to be 11.2 × 10^10^ gC year^−1^ and the DIC efflux from the bay to be 13.4 × 10^10^ gC year^−1^ 13. By combining these budget estimates, an additional carbon input of 7.7 × 10^10^ gC year^−1^ will be required, a value roughly equal to our estimate of net CO_2_ uptake. In addition, the above analysis also suggests that the eventual sink of the fixed carbon from CO_2_ absorption in Tokyo Bay would be both from its burial in bay sediments and export to outer oceanic areas.

Because our observations were conducted on monthly basis, our estimates of CO_2_ flux may have failed to report on events on a daily and/or weekly time-scale. We acknowledge that our results regarding the net CO_2_ uptake in the bay might be compromised if these short-term temporal events contributed to significant CO_2_ emission or outgassing from surface waters. We postulated two types of outgassing events in Tokyo Bay and found that such outgassing events should be insignificant to our carbon budget. First, we postulated a sudden vertical mixing event in early autumn when stratification was weakened. The observed pCO_2_ values of the bottom waters during the stratification season from June to September were 400–940 μatm^7^, with a typical water-column average of approximately 600 μatm. Even if the outgassing from seawater at this level of CO_2_ occurred in the entire area of the bay for 30 days, the amount of CO_2_ efflux under typical wind speed would be 0.6 × 10^10^ gC, which would account for 12% of the air-sea CO_2_ exchange in the bay (5.2 × 10^10^ gC). Second, we postulated coastal upwelling events, which are generally observed in the northeastern part of the bay in late summer. These upwelling events cause conspicuous milky turquoise waters^14^ due to the oxidation of hydrogen sulfide in anoxic bottom waters. We observed these bottom waters on 16 and 23 September, 2010, in which pCO_2_ values ranged from 765 to 1,161 μatm with an average of 967 μatm and the average CO_2_ flux was estimated to be 54.0 mmolC m^−2 ^day^−1^. Because of the conspicuous water color, the area and duration of the upwelling events are well described in the bay; the events were observed for an average of 11.4 days a year from 2007 to 2010, with a maximum area of 80 km^2^^15^. On the basis of this average duration and area, the observed CO_2_ flux would yield an annual flux of 5.9 × 10^8^ gC year^−1^. This value accounts for only 1.2% of the air-sea CO_2_ exchange in the entire bay.

**Figure 4:**
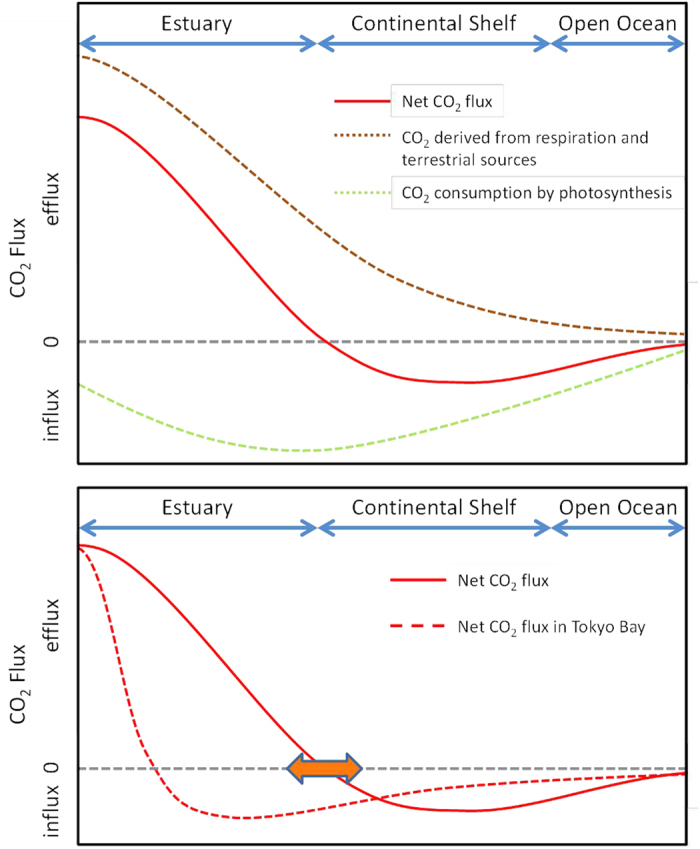
Conceptual scheme of net CO_2_ flux in coastal waters and open ocean. The upper panel shows net CO_2_ flux (red line), which is estimated from the “CO_2_ derived from respiration and terrestrial sources (dotted brown line)” plus the “CO_2_ consumption by photosynthesis (dotted green line)” The lower panel shows net CO_2_ flux in Tokyo Bay (dotted red line). The dotted gray line denotes the atmospheric CO_2_ equilibrium (air-sea CO_2_ flux is ±0). The orange arrow indicates the border shift where air-sea CO_2_ flux is ±0 between the nearshore waters emitting CO_2_ and outer waters absorbing CO_2_. In the case of Tokyo Bay, the border is extremely shifted inshore because of the low organic carbon supply and expansive coverage of secondary sewage treatment plants (STPs) in the catchment area.

This study clearly demonstrated that Tokyo Bay as a whole is a net sink for atmospheric CO_2_; however, this finding may contradict those of many studies on coastal waters. Most inland waters and estuaries have been reported to be significant sources of CO_2_ to the atmosphere due to respiration of terrestrial organic carbon^2^,^3^,^16^ and terrestrial input of freshwater CO_2_^17^. In contrast, continental shelf areas, which are laid offshore of coastal areas, have generally been reported to be sinks for atmospheric CO_2_ due to nutrient input through coastal waters and from pelagic deep waters^2^,^3^,^18^,^19^. Oceanic basins that are further offshore are either weak sinks or weak sources of atmospheric CO_2_, depending on the biogeochemical settings of the basin^20^. Although it is confined to a very small inner part of the bay, Tokyo Bay certainly has an area of CO_2_ emission, and the CO_2_ emission mechanism in this area is common to that of other coastal areas. The net CO_2_ absorption in the main body of the bay is driven either by a mechanism similar to that of continental shelf regions or by biological CO_2_ fixation with a terrestrial supply of nutrients. On the basis of these considerations, we propose a generalized scheme for the CO_2_ budget in a continuing water system composed of nearshore water emitting CO_2_, an outer water absorbing CO_2_, and pelagic water with a neutral CO_2_ budget (Fig. 4).

In this scheme, there would be a border where the air-sea CO_2_ flux is ±0 between the nearshore waters emitting CO_2_ and an outer waters absorbing CO_2_. On the land-side of this border, CO_2_ emission due to biological degradation of terrestrial organic matter would exceed CO_2_ uptake due to photosynthesis. The CO_2_ emission would decrease toward the offshore side, and photosynthetic CO_2_ uptake would exceed emissions on the offshore side of this border. The location of this border may shift either offshore or inshore. In fact, several studies have observed that some waters in estuaries with small freshwater discharge are weak sources or sometimes weak sinks of CO_2_^21^,^22^,^23^. In addition, some continental shelves with large freshwater discharge have been reported to be sources of CO_2_ to the atmosphere^19^. We interpret that these rather atypical observations are associated with the shift of the aforementioned border. The shift is likely caused by the different terrestrial organic carbon load accompanied by freshwater discharge^24^. Other factors that may affect the border shift and intensity of CO_2_ flux include hydrographic and geomorphological characteristics such as a stratified estuarine system^22^, a microtidal estuarine system^25^, an open/enclosed nature of the coast^23^,^26^, and a submerged aquatic vegetation in shallow coastal waters^27^.

In the case of Tokyo Bay, the border is extremely shifted inshore. The shift reflects the relatively low organic carbon supply from land and the active organic matter production driven by the massive nutrient supply from land. The seasonal stratification and semi-enclosed nature of the embayment should further facilitate the net uptake of CO_2_ in Tokyo Bay. The low organic matter supply and large nutrient supply from land may seem contradictory. We believe that this imbalance between organic matter and nutrient supply is largely derived from the secondary sewage treatment in the catchment area of Tokyo Bay^4^. Currently, secondary-treated effluent flowing into Tokyo Bay accounts for 50% of the total freshwater discharge^28^. Sewage treatment plants along Tokyo Bay remove organic carbon from freshwater at a rate of 7.7 × 10^10^ gC year^−1^ 28. This value is comparable to the uptake of CO_2_ observed in this study (5.2 × 10^10^ gC year^−1^). As the quantity of dissolved organic carbon (DOC) flowing into the bay has decreased by 60% in the last 40 years, the quality of DOC has become more recalcitrant due to improved sewage treatment^29^. The decrease in turbidity due to the decrease in organic carbon inflow has also enhanced phytoplankton activity from the improvement of light availability^30^. The effect of sewage treatment has also been reported in other estuary systems; Amann *et al*.^31^ have recently reported that pCO_2_ decreased from 7000 to 2500 μatm at the oxygen minimum zone in 1986 and 2007 due to the installation of sewage treatment plants in the Elbe estuary.

Urbanized coastal waters that have a relatively large coverage of secondary sewage treatment tend to act as a strong net sink for atmospheric CO_2_. The progress made in the urbanization of coastal areas with improved sewage treatment is common in many parts of the world^32^. This development will have an impact on the future budget of marine CO_2_. Moreover, approximately 40% of the world’s population is currently settled in coastal zones, and developed urban areas with completed sewer systems are expected to expand rapidly^32^. According to a UNEP report^32^, highly developed coastal areas occupied only 15% of the world coastal zones in 2002, but this figure is expected to rise to 60% in 2050. Therefore, the CO_2_ budget characteristic of Tokyo Bay is expected to be observed in more marine coastal areas in the future. Although it may be difficult to predict future trends, we assume that highly urbanized coastal waters currently occupy 15% of the world coastal zones and are expected to occupy 60% by 2050. When the CO_2_ fluxes in highly urbanized coastal waters and ordinal coastal waters are the same values as that of Tokyo Bay (–3.2 molC m^−2^ year^−1^) and previously published data^3^ (7.7 molC m^−2^ year^−1^), respectively, the CO_2_ emissions from global coastal waters will be estimated to be approximately 0.074 PgC year^−1^ at present and 0.014 PgC year^−1^ in 2050. The emission of CO_2_ to the atmosphere from estuaries is calculated to be 21% and 85% lower than previous estimates. As a result, the CO_2_ budget of the global coastal waters would be expected to be a sink rather than a source.

## Methods

The ship routes of all cruises were presented in Fig. 3 (a). Each point in this figure represents a pCO_2_ value measured at a one-minute interval. Observations of pCO_2_, salinity, and temperature were conducted during 40 cruises of the R/V Seiyo-maru and 9 cruises of the R/V Hiyodori. Measurements of pCO_2_, salinity, and temperature were taken with a sampling frequency of one minute. Surface seawater was pumped up from the ship’s bottom at ca. 2-m depth. Our pCO_2_ measuring system consisted of a NDIR analyzer (LI-820, Li-Cor) and a membrane equilibrator. The membrane equilibrator was composed of multi-layered composite hollow-fiber membrane modules^33^ (MHF module, Mitsubisi Rayon Co., Ltd.). The equilibrator was made of 6 MHF modules to create more surface area and a more rapid response. The response time and the standard error of this system were approximately 100 seconds, and smaller than 0.4 μatm, respectively. Atmospheric pCO_2_ (pCO_2_^air^) was measured every three hours. *In situ* surface water salinity and temperature were measured using a thermosalinograph (Tsurumi Seiki Co., Ltd. at Seiyo-maru and YSI 6920 at Hiyodori). The net flux of CO_2_ across the air-sea interface was calculated as the product of the solubility of CO_2_ in seawater^34^, the gas transfer piston velocity of CO_2_, and the difference between pCO_2_^sea^ and pCO_2_^air^. The gas transfer piston velocity was calculated using the equation given by Wanninkhof 35. Wind speed data were obtained from the Japan Coast Guard (http://www6.kaiho.mlit.go.jp/tokyowan/) from stations (Tsurugisaki, Kannonzaki, Honmoku, and Tokyo 13 gouchi) close to the observation areas. The samples for chlorophyll *a* (Chl *a*) measurement were collected at each stations using bucket and filtered through precombusted (450 °C, 3 h) GF/F filters. After filtration, chlorophyllous pigments were extracted using N, N-dimethylformamide, and the concentrations of Chl a were determined by the fluorometric method^36^ (fluorometer used TD-700, Turner Desings).

## Additional Information

**How to cite this article**: Kubo, A. *et al*. A significant net sink for CO_2_ in Tokyo Bay. *Sci. Rep.* **7**, 44355; doi: 10.1038/srep44355 (2017).

**Publisher's note:** Springer Nature remains neutral with regard to jurisdictional claims in published maps and institutional affiliations.
